# Allergic Airway Inflammation by Nasal Inoculation of Particulate Matter (PM_2.5_) in NC/Nga Mice

**DOI:** 10.1371/journal.pone.0092710

**Published:** 2014-03-26

**Authors:** Keiki Ogino, Ran Zhang, Hidekazu Takahashi, Kei Takemoto, Masayuki Kubo, Ikuo Murakami, Da-Hong Wang, Yoshihisa Fujikura

**Affiliations:** 1 Department of Public Health, Okayama University Graduate School of Medicine, Dentistry and Pharmaceutical Sciences, Okayama, Japan; 2 Department of Public Health, Yamaguchi University Graduate School of Medicine, Ube, Yamaguchi, Japan; 3 Third Institute of New Drug Discovery, Otsuka Pharmaceutical Co., Ltd., 463-10 Kagasuno, Kawauchi-cho, Tokushima, Japan; 4 Department of Molecular Anatomy, Oita University Faculty of Medicine, 1–1, Idaigaoka, Hasama-machi, Yufu, Oita, Japan; National Jewish Health, United States of America

## Abstract

To evaluate the effect of airborne particulate matter 2.5 (PM_2.5_) in winter on airway inflammation, water-soluble supernatant (Sup) and water-insoluble precipitate (Pre) in PM_2.5_ were inoculated in NC/Nga mice with high sensitivity to mite allergens. Sup with aluminum oxide was injected intraperitoneally for sensitization. Five days later, Sup, Pre or both Sup and Pre were inoculated via the nasal route five times for more sensitization and a challenge inoculation on the 11th day in NC/Nga mice. On the 12th day, mice were examined for airway hyperresponsiveness (AHR), BALF cell count and IL-1β concentration, mRNA expression of Th_1_ and Th_2_ cytokines, chemokines such as eotaxin 1 and eotaxin 2, inflammasomal complex molecules such as IL-1β, caspase 1 and the nucleotide-binding domain and leucine-rich repeat protein 3 (NLRP3) in lung tissue as well as histopathology. The synergistic effect of Sup and Pre was observed in terms of increases in AHR, BALF cells, the mRNA expression of IL-13, eotaxin1 and IL-1β, and the IL-1β concentration in BALF. Intracellular deposits of insoluble particulates were observed in macrophages around inflammatory granulation of the mouse group treated with Sup and Pre. These results suggest that PM_2.5_ can induce airway hyperresponsiveness in mice with genetically high sensitivity to mite allergens by an inflammasome-associated mechanism and synergistic action of insoluble particulates and soluble components.

## Introduction

Recently, much attention has been focused internationally on the environmental problems associated with PM from China. Many people in China have asthma induced by a high level of PM_2.5_. Particles of various sizes exist in the atmosphere as particulate matter (PM). In particular, PM, with a particle size of 10 microns (PM_10_) or 2.5 microns (PM_2.5_), has been focused on owing to its entry into the bronchus. Many epidemiological studies have revealed the associations between PM and the respiratory system [1], [2], [3]. Some biological agents such as endotoxin, β-glucan and mold spores [4], diesel exhaust particles (DEP) [5], [6], metals [7], [8], [9] and ultrafine particles adhered to coarse particles [10] in PM are considered to be constituents responsible for the inflammatory and toxic effects on the respiratory system, although specific components responsible for adverse effects have not been fully investigated. For respiratory allergic reactions in particular, DEP [11] and metals in residual oil fly ash [12] are said to be involved. Moreover, ambient urban Baltimore particulates induced allergic airway reaction in mice [13]. However, among these allergy-related PM components, there was no evidence to demonstrate the contribution of soluble protein to the PM-induced airway allergic reaction, although almost all PM-related allergic reactions occurred with the aid of ovalbumin (OVA) or mite allergens as an adjuvant. We previously demonstrated for the first time that total suspended matter could induce airway inflammation with AHR by the action of soluble protein and insoluble precipitate [14].

Airway inflammation with the recruitment of Th2 lymphocytes is a prerequisite for asthma. Th2 cytokines such as IL-4, IL-5 and IL-13 play critical roles in allergic disorders [15]. Previously, we established a new experimental asthma model in NC/Nga mice with intranasal mite allergen exposure [16]. Instead of the commonly used animal model of asthma induced by OVA, our mouse model showing increased eosinophils and high expression of IL-4, IL-5 and IL-13 detected in BALF and lung tissue is considered to bear a closer resemblance to human asthma [16], [17], [18]. Moreover, it has been shown that NC/Nga mice have high sensitivity to mite allergen not only in terms of airway inflammation but also atopic dermatitis [19], [20], [21].

Various stimulants such as lipopolysaccharide (LPS) [22] and exposure to silica and asbestos [23], as well as intracellular danger signals such as reactive oxygen species (ROS) [23], ATP [24] and uric acid crystals [25], transfer signals to the inflammasome protein complex consisting of the nucleotide-binding domain and leucine-rich repeat protein 3 (NLRP3), adaptor protein apoptosis-associated speck-like protein (ASC) and inactive caspase-1. Activation of caspase-1 by its autocleavage leads to the molecular change of immature pro-IL-1β to mature active IL-1β [26]. Secreted active IL-1β is said to be associated with atherosclerosis, diabetes, obesity, gout and autoimmune disease [27]. PM10 was shown to induce inflammasome-associated IL-1β secretion in a human monocyte cell line [28] and airway epithelium in mice [29]. Immunohistochemical localization and induction of NLRP3 inflammasome were also demonstrated in an ovalbumin-induced asthma model [30].

In this study, we demonstrated for the first time that PM_2.5_ could induce airway hyperresponsiveness in NC/Nga mice, which are potentially hypersensitive to mite allergens. Therefore, we explored the mechanisms for airway reaction by the effect of insoluble particles and the soluble element of PM_2.5_, considering the involvement of inflammasome.

## Materials and Methods

### Animals

Pathogen-free male (7-wk-old) NC/Nga mice were obtained from Charles River Laboratories Japan (Yokohama, Japan). All mice were housed in a specific pathogen-free environment with a 12-h light and 12-h dark cycle. The mice were provided with water and food ad libitum. The care and handling of the animals were in accordance with the Guidelines for the Care and Use of Laboratory Animals at Shikata Campus of Okayama University. This study was approved by the Okayama University Institutional Animal Care and Use Committee (approval number: OKU-2012467).

### Chemicals

Oligonucleotide primers for real-time PCR were synthesized by TAKARA BIO (Otsu, Shiga, Japan). All other chemicals were of the highest quality that was commercially available.

### Source of PM_2.5_

Airborne particulate matter, less than 2.5 μm in aerodynamic diameter (PM_2.5_), was collected with an Anderson-type high-volume sampler (HV-1000R, Shibata, Japan) using a quartz filter (8 cm×10 cm, 2500QAT-UP, Pallflex Products, Putnam, CT, USA) at a flow rate of 166 L min^−1^ continuously for 7 days and 4 filters were collected from January 30 to March 14 in Okayama city. This flow rate was aerodynamically calculated to obtain airborne particulate matter of less than 2.5 μm in aerodynamic diameter, in the backup filter. The used filters were stored in a freezer (−30°C) until pretreatment.

### Separation of Particulate Matter Fractions

Four quartz fiber filters treated with Teflon were cut and immersed in 2 mM phosphate buffer (PBS), pH 7.4, prepared with pyrogen-free water, and left to stand for 20 min. Contaminated quartz fibers were filtrated with a 5 μm syringe filter (Minisart; Sartorious Stedim Biotech GmbH, Goettingen, Germany) and the filtrate was concentrated by vacuum freeze-drying. Water was added to the freeze-dried product, which was then centrifuged at 5000 rpm. The soluble supernatant (Sup) in water was adjusted in terms of its protein concentration. The weight of the precipitated water-insoluble fraction (Pre) was measured after vacuum drying and it was then suspended in physiological saline solution or protein-containing supernatant.

### Measurement of Polynuclear Aromatic Hydrocarbons by GC-MS

Polynuclear aromatic hydrocarbons (PAHs) were eluted from insoluble Pre using toluene [14]. The determination of PAHs was carried out using a 7890A gas chromatograph equipped with a 5975C inert XL mass spectrometer (GC-MS, Agilent Technologies, Palo Alto, CA, USA). The column was a 30 m×0.25 mm ID DB-5 MS capillary column with 0.25-mm film thickness (Agilent Technologies, Palo Alto, CA, USA). Helium was used as the carrier gas at a flow rate of 1.2 ml/min. The temperatures of the injection port and the transfer line were set at 300°C. The oven temperature was set at 75°C for 3 min and then increased to 250°C at a rate of 25°C/min, and to 300°C at a rate of 5°C/min. Samples (1 ml) were injected in pulsed split mode (split ratio: 10∶1, pulse pressure: 25 psi, pulse time: 1 min). The mass spectrometer was operated in the electron impact (EI) mode at an electron energy of 70 eV. The ion source and quadrupole analyzer were maintained at 230 and 150°C, respectively. Data were obtained in scan mode with the scan ranging from m/z 40 to 450. EPA 610 Polynuclear Aromatic Hydrocarbons Mix (Supelco Inc., Bellefonte, PA, USA) was used as a standard for PAHs.

### Measurement of Metals by ICPM

Metals of Sup and Pre were digested in a collision cell using an Ethos 900 Milestone Digester (Milestone General K.K., Kawasaki, Japan), in addition to nitric acid, hydrogen peroxide and hydrofluoric acid, and digested metals were quantitatively measured using an Inductively Coupled Plasma Mass Spectrometer (ICPM-8500, Shimadzu, Tokyo, Japan). Metals in PM2.5 were represented by their values in Sup and their values in Pre per g Pre.

### Measurement of Protein

Soluble Sup and insoluble Pre suspended in TBS containing 2% sodium dodecyl sulfate (SDS) were measured for protein content using the BCA method.

### Administration to Animals

Supernatant solution containing 50 μg of protein (Sup) was mixed with 4 mg of aluminum hydroxide and administered intraperitoneally in mice for sensitization. Sample solution containing 200 μg of precipitate (Pre) in 25 μl of sterile phosphate buffer (PB) (20 mM, pH 7.4) and supernatant solution containing 50 μg of protein (Sup) or 200 μg of precipitate suspended in 25 μl of supernatant solution containing 50 μg of protein (Sup plus Pre) was administered to NC/Nga mice by intranasal instillation on 5 consecutive days (experimental days 6–10) and on day 15 (single challenge) under anesthesia with pentobarbital, as previously described [16]. For the control group, 25 μl of physiological saline was administered five times for sensitization and a time challenge by successive nasal inoculation. Serum and whole lung tissue were collected for further analysis.

### Analysis of Cells and IL-1β in the BALF

Bronchoalveolar lavage fluid (BALF) was collected with 1.0 ml of saline after death using a tracheal cannula [17]. After centrifugation, cell pellets were resuspended in 1 ml of PBS, and total BALF cell counts were determined with a hemocytometer. BALF cytospins were prepared, slides were fixed in acetone and then Wright-Giemsa staining was performed. A blinded observer determined the percentages of eosinophils, neutrophils and mononuclear cells on each slide by counting a minimum of 200 cells in random high-power fields with a light microscope.

The supernatant of BALF was measured with IL-1β using an ELISA kit (R & D Systems Inc., MN, USA).

### Airway Hyperresponsiveness (AHR)

On day 15, the degree of bronchoconstriction was measured according to the overflow method [16]. Briefly, mice were anesthetized with pentobarbital (80 μg/kg) and connected to an artificial ventilator following surgical incision of the trachea. A pulmotor system was constructed with a rodent ventilator (Model 132; New England Medical Instrument Inc., Medway, MA), a bronchospasm transducer (Model 7020; Ugo Basile, Comerio-Barese, Italy) and a data recorder (Omniace II data acquisition system, Model RA1300; NEC San-ei, Tokyo, Japan). Gallamine triethiodide (350 μg/mouse) was intravenously administered immediately to eliminate spontaneous respiration, followed by the administration of acetylcholine with stepwise increases in the concentration from 62.5 to 4000 μg/kg. Dose-response curves for acetylcholine in anesthetized, mechanically ventilated mice were obtained. Bronchoconstriction is expressed as the respiratory overflow volume provoked by acetylcholine as a percentage of the maximal overflow volume (100%) obtained by totally occluding the tracheal cannula [31].

### Real-time PCR for Cytokines in the Lung Tissue

Total RNA was purified from approximately 100 mg each of lung tissue by liquid phase separation using ISOGEN (NIPPON GENE) in combination with High-Salt Precipitation Solution (NIPPON GENE). The RNA concentrations were measured on a NanoDrop 1000 spectrophotometer (NanoDrop Technologies). The integrity of RNA was verified by denaturing agarose gel electrophoresis. The complementary DNA (cDNA) was synthesized from 1 μg each of total RNA in a 20 μl reaction volume using PrimeScript 1st strand cDNA Synthesis Kit (Takara 6110) with the Oligo dT primer according to the manufacturer’s instructions. An aliquot of cDNA was used as a template for quantitative PCR using SYBR Premix Ex Taq (Tli RNaseH Plus) (Takara RR420) with the ROX dye passive reference on StepOnePlus Real-Time PCR Systems (Applied Biosystems; the Central Research Laboratory, Okayama University Medical School) operated in the relative gene expression mode using the ROX dye as a passive reference. The primers used are listed in Table 1. The primers were chosen from either PrimerBank [32] or qPrimerDepot [33], such that each amplicon spans at least one intron. The relative expression levels were calculated by the ΔΔC_T_ method using the GAPDH gene as an endogenous control for normalization, with the following modification: the mean PCR amplification efficiency for each primer set was calculated by carrying out the PCRs in duplicate using undiluted and 4-fold-diluted cDNA as templates. The expression level in each sample represents the geometric mean of the duplicate measurement with the dilution rate and the amplification efficiency taken into account. In a few samples where multiple peaks were observed in the melting temperature curve of one of the duplicate measurements, the expression level of only one of the duplicates with a single peak was represented. Finally, the geometric mean of the relative expression level in the saline-administered group was set to 1.

**Table 1:** Primers used for qRT-PCR.

Target gene	Forward primer (5′ to 3′)	Reverse primer (5′ to 3′)	Source
GAPDH	AGGTCGGTGTGAACGGATTTG	TGTAGACCATGTAGTTGAGGTCA	PrimerBank
Eotaxin-1 (Ccl11)	GAATCACCAACAACAGATGCAC	ATCCTGGACCCACTTCTTCTT	PrimerBank
Eotaxin-2 (Ccl24)	AATTCCAGAAAACCGAGTGG	TCTTATGGCCCTTCTTGGTG	qPrimerDepot
IL-1β	GCAACTGTTCCTGAACTCAACT	ATCTTTTGGGGTCCGTCAACT	PrimerBank
IL-4	TGAACGAGGTCACAGGAGAA	CGAGCTCACTCTCTGTGGTG	qPrimerDepot
IL-5	CTCTGTTGACAAGCAATGAGACG	TCTTCAGTATGTCTAGCCCCTG	PrimerBank
IL-13	TGTGTCTCTCCCTCTGACCC	CACACTCCATACCATGCTGC	qPrimerDepot
IFN-γ	ATGAACGCTACACACTGCATC	CCATCCTTTTGCCAGTTCCTC	PrimerBank
TNF-a	CCACCACGCTCTTCTGTCTAC	AGGGTCTGGGCCATAGAACT	qPrimerDepot
Caspase 1	TCAGCTCCATCAGCTGAAAC	AGTCCTGGAAATGTGCCATC	qPrimerDepot
NLRP3	AAAATGCCTTGGGAGACTCA	AAGTAAGGCCGGAATTCACC	qPrimerDepot
GAPDH	AGGTCGGTGTGAACGGATTTG	TGTAGACCATGTAGTTGAGGTCA	PrimerBank

### Histological Experiments

After exsanguination, the lungs were cut, fixed with 10% neutral phosphate-buffered formalin and embedded in paraffin. The fixed lung tissues were dehydrated, embedded in paraffin and sectioned. Hematoxylin and eosin (H&E) staining and periodic acid-Schiff (PAS) staining were used for the inflammatory changes of the lung tissue and goblet cell hyperplasia.

### Statistical Analysis

The results are presented as means ± SEM. Data were analyzed by one-way ANOVA or two-way ANOVA to examine whether there were any significant differences among groups. If the difference evaluated by ANOVA was significant, Bonferroni post hoc tests or Tukey’s multiple comparison tests were used for paired comparisons. A two-tailed unpaired t-test was used for comparison with control values, using GraphPad Prism 5.0c for Mac (GraphPad Software, Inc., San Diego, CA). A *p* value less than 0.05 was considered statistically significant.

## Results

### PM Characterization

Metals and PAHs in PM_2.5_ were measured (Table 2). Ambient PM_2.5_ contained high levels of zinc, lead and aluminum. In terms of organic components of PM_2.5_, high levels of naphthalene, fluoranthene, benzo(b)fluoranthracene and indeno(1, 2, 3-cd)pyrene were detected.

**Table 2:** Metal and organic compound concentrations in PM2.5.

Metal	Concentrations (μg/g)	Organic compounds	Concentrations (μg/g)
Aluminum	1100.0	Anthracene	ND
Arsenic	322.9	Benzo(a)anthracene	8.9
Beryllium	ND	Benzo(b)fluoranthene	29.6
Cadmium	17.1	Dibenzo(a, h)anthracene	8.0
Chromium	102.6	Benzo(k)fluoranthene	15.6
Copper	316.4	Benzo(a)pyrene	17.0
Iron	ND	Chrysene	13.5
Lead	876.6	Dibenzo(a, h)anthracene	8.0
Manganese	687.9	Fluoranthene	9.6
Nickel	201.6	Fluorene	4.9
Selenium	192.9	Indeno(1,2,3-cd)pyrene	32.7
Uranium	ND	Naphthalene	289.2
Vanadium	299.2	Phenanthrene	14.2
Zinc	2427.4	Pyrene	10.4

The protein content of Sup was 11.4 mg per 1.5 ml. Collected PM was 34.6 mg. By calculation, 0.1 mg of protein was released from Pre per mg.

### Effect of PM on AHR

**Figure 1:**
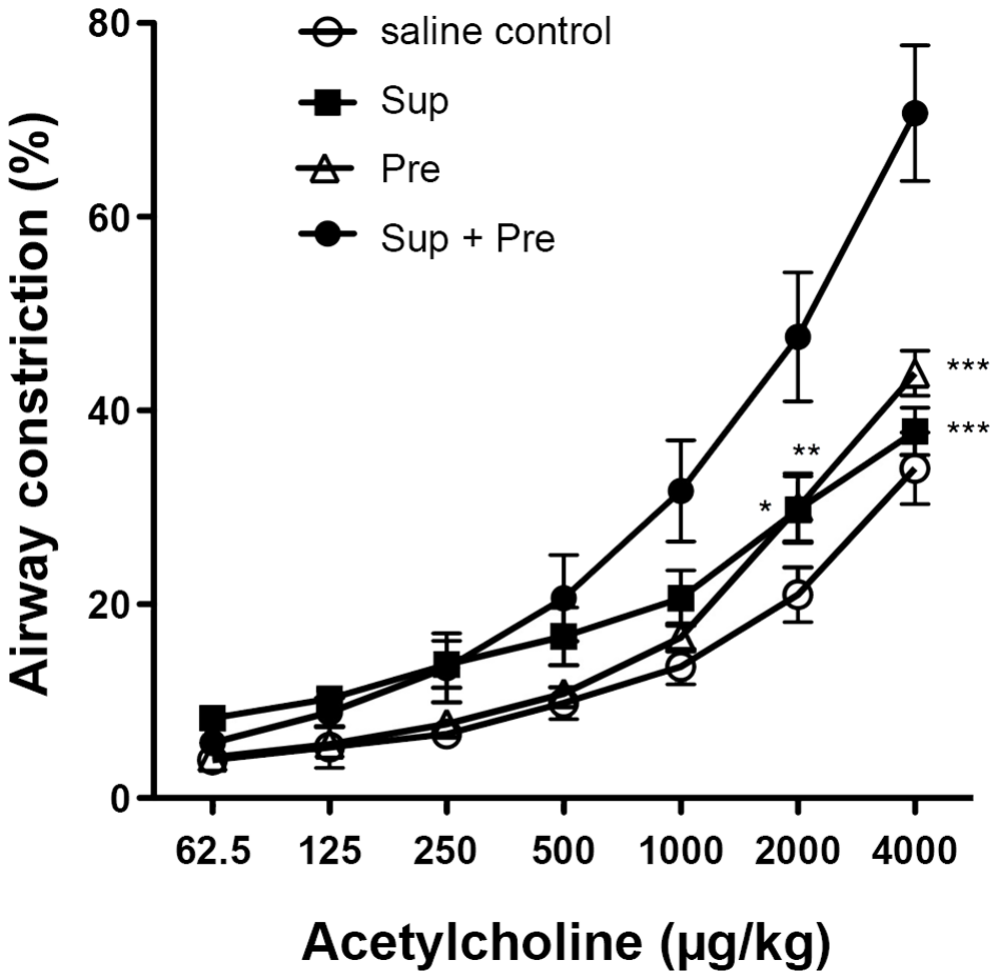
AHR to acetylcholine after exposure to PM. Effect of control (sterilized physiological saline), Sup (supernatant fraction containing 50 μg of protein), Pre (200 μg of precipitate in 25 μl of PB) and Sup plus Pre administered intranasally in NC/Nga mice. The bronchoconstriction (%) is expressed as mean ± SEM of 5–8 animals (n = 8, 7, 5 and 5 for saline control, Sup, Pre and Sup+Pre groups, respectively). Two-way ANOVA showed P<0.0001 concerning responses to acetylcholine and mouse groups. *P<0.05, **P<0.01 and ***P<0.001 represent multiple-comparison test results of the dose responses to acetylcholine in the mouse groups of Sup, Pre and control vs. the mouse group of Sup plus Pre.

Bronchoconstriction values for AHR were not significantly different among the treatment groups of saline control, Sup and Pre. However, bronchoconstriction values of Sup plus Pre against 2,000 μg/kg and 4,000 μg/kg acetylcholine were significantly higher than those of the other treatment groups (Figure 1). In particular, synergistic action of AHR was observed in Sup plus Pre compared with Sup and Pre.

### Effect of PM on BALF Cell Analysis and IL-1β Secretion

**Figure 2:**
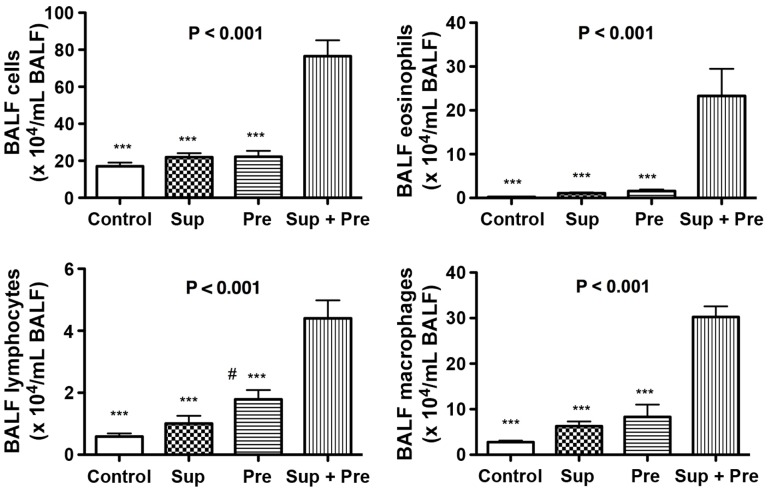
BALF cell numbers of total cell fraction, eosinophils, lymphocytes and macrophages. The counts of these cells in the BALF are expressed as mean ± SEM of 4–8 animals (n = 8, 6, 4 and 4 for Control, Sup, Pre and Sup+Pre groups, respectively). P for ANOVA is expressed in the upper portion of each figure. Tukey’s multiple-comparison test results are shown in the mouse groups of control, Sup and Pre compared with the mouse group of Sup plus Pre as ***P<0.001 and the Pre group compared with the control as # P<0.05.

Total cell numbers, eosinophils, lymphocytes and macrophages in BALF were significantly increased in mice inoculated with Sup plus Pre compared with those of mice exposed to the control. Moreover, the synergistic up-regulation of BALF cells was also observed in mice with Sup and Pre compared with the levels in Sup and Pre (Figure 2).

**Figure 3:**
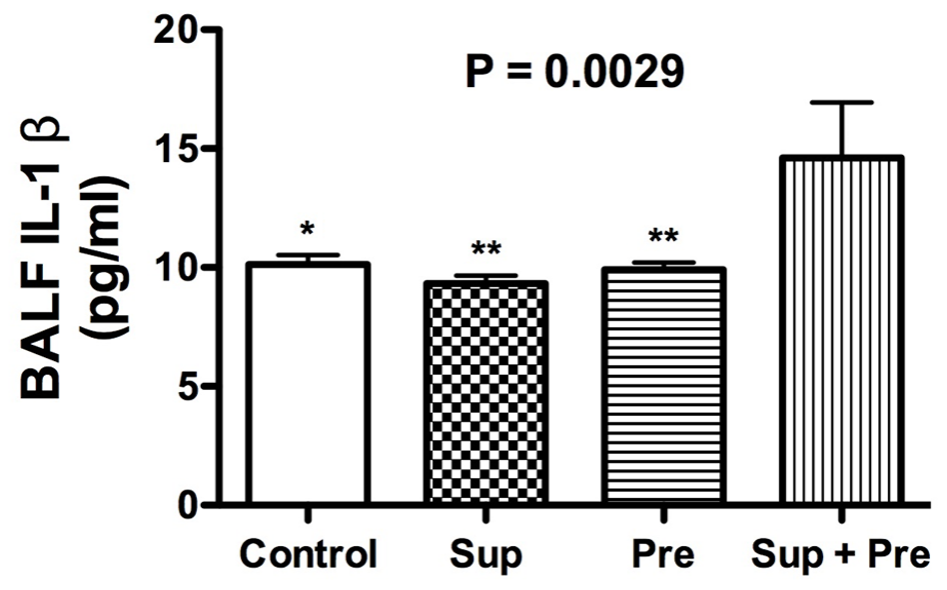
IL-1β concentration of BALF was determined using ELISA. Data are expressed as the mean ± SEM from 5–8 mice (n = 8, 7, 8 and 5 for Control, Sup, Pre and Sup+Pre groups, respectively). P for ANOVA is expressed in the upper portion of each figure. *P<0.05 and **P<0.01 represent Tukey’s multiple-comparison test results of each group compared with Sup plus Pre.

IL-1β in BALF was measured using ELISA. The IL-1β concentration in BALF was significantly increased in mice exposed to Sup plus Pre compared with that with PB control, Sup and Pre (Figure 3).

### Effect of PM on Expression of mRNA for Cytokines in the Lung Tissue

**Figure 4:**
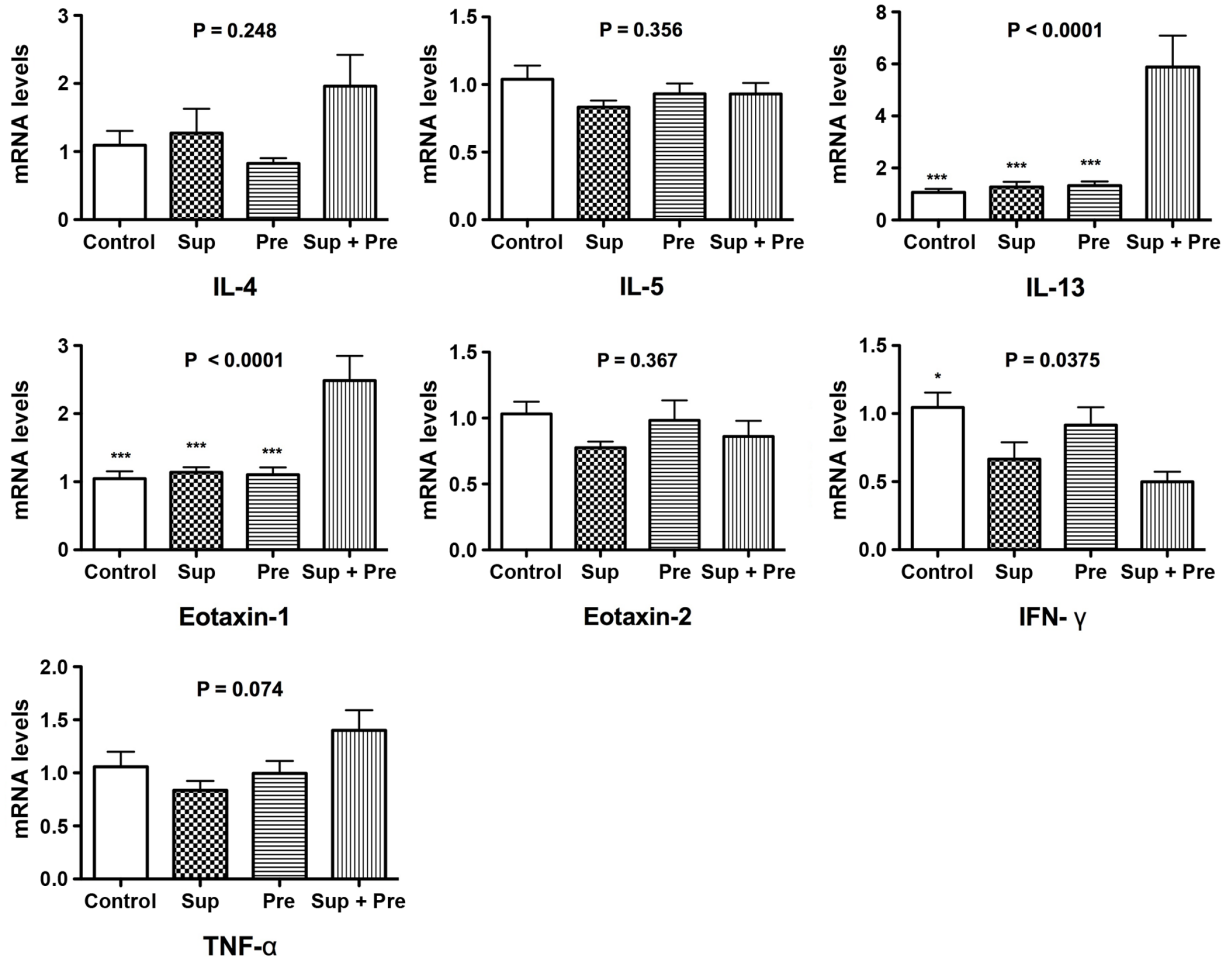
Changes in mRNA expression for cytokines and chemokines in the lung tissue after exposure to PM. Mice were administered saline control, supernatant (Sup) containing 50 μg of protein, 200 μg of precipitate (Pre) and Sup plus Pre. Real-time PCR for the cytokines IL-4, IL-5, IL-13, eotaxin-1, eotaxin-2, IFN-γ and TNF-α was performed under optimized conditions. Relative expression was calculated with GAPDH as an internal standard. Data are expressed as the means ± SEM from 5–8 mice (n = 8, 7, 8 and 5 for Control, Sup, Pre and Sup+Pre groups, respectively). P for ANOVA is expressed in the upper portion of each figure. *P<0.05 and ***P<0.001 represent Tukey’s multiple-comparison test of control, Sup and Pre compared with Sup plus Pre.

**Figure 5:**
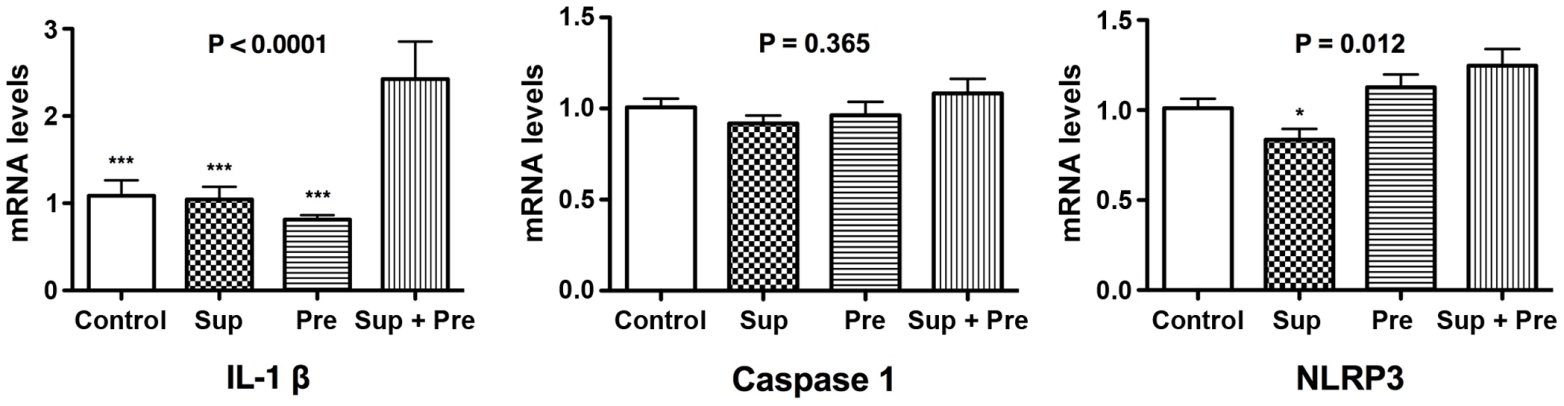
Changes in mRNA expression for IL-1β, caspase 1 and NLRP3 in the lung tissue after exposure to PM. Data are expressed as the mean ± SEM from 5–8 mice (n = 8, 7, 8 and 5 for Control, Sup, Pre and Sup+Pre groups, respectively). P for ANOVA is expressed in the upper portion of each figure. *P<0.05 and ***P<0.001 represent Tukey’s multiple-comparison test of each group compared with Sup plus Pre.

The expression of mRNA of several cytokines (Figure 4) and inflammasome protein complex (Figure 5) was evaluated by PCR. Prominent synergistic up-regulation, approximately 6 times higher than that of Sup and Pre, was observed for the mRNA of IL-13 in mice exposed to Sup plus Pre. Synergistic up-regulation of mRNA for eotaxin 1 and IL-1β was also detected in mice exposed to Sup plus Pre, compared with Sup and Pre. However, there was no difference in mRNA levels of IL-4, IL-5, eotaxin 2, TNFα and caspase 1 among mice exposed to saline control, Sup, Pre and Sup plus Pre. Moreover, decreases in mRNA levels of interferon (IFN)-γ and NLRP3 were observed in the mouse group of Sup plus Pre compared with that of the PB control and in the mouse group of Sup compared with that of Sup plus Pre.

### Effect of PM on Histopathological Changes of the Lung

**Figure 6:**
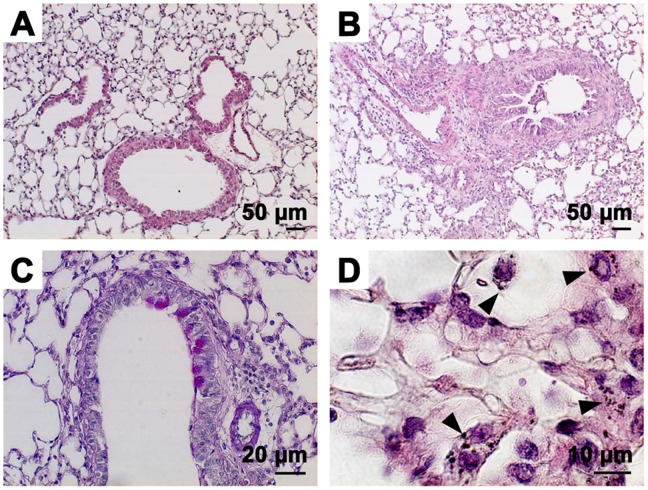
Histopathological findings of the lungs after exposure to PM. Paraffin-embedded sections were stained with hematoxylin-eosin for the saline control (A) and Sup plus Pre (B). The periodic acid-Schiff (PAS) staining of Sup plus Pre mice is shown in C. High magnification of H&E staining of the site of inflammatory granulation of Sup plus Pre mice (D).

Peribronchial inflammation was observed in the lungs of Sup plus Pre-inoculated mice (Figure 6B) compared with no inflammation in saline control mice (Figure 6A). However, PAS-positive epithelial cells were not so abundant in Sup plus Pre-inoculated mice (Figure 6C). Many PM particles shown by arrowheads were observed inside inflammatory macrophage-like cells (Figure 6D).

## Discussion

This study is the first to report that PM_2.5_ could evoke allergic airway inflammation in mice. In particular, the synergistic action of Sup and Pre, components of PM_2.5_, can elicit airway inflammation by the elevation of eosinophils in BALF, Th2-type immunoresponse and the activation of inflammasome such as IL-1β.

The synergistic action of the soluble fraction and the insoluble fraction was a prerequisite for asthma-like airway allergic reaction in this study. In PM_2.5_, 0.022 mg of protein was contained per mg of insoluble PM and a total of 0.47 mg of protein was considered to be contained per mg of PM_2.5_ by the addition of protein content in soluble Sup to protein content in insoluble Pre. Ambient urban Baltimore PM was previously shown to include 0.015 mg of protein per mg PM [13]. Compared with this, a similar level of protein content was observed in PM in our study. However, little evidence on the protein of PM has been reported. In our previous study, the attached protein component in suspended particles (TSP) contributed to asthma-like airway inflammation in NC/Nga mice and synergistic action of Sup and Pre for AHR was observed [14]. Moreover, originally, NC/Nga mice were shown to develop allergic reactions to mite allergens easily, which resulted in atopic-like dermatitis [18], [19], [20] and asthma [15], [16], [17]. Therefore, some aeroallergens may be involved in this airway allergic reaction because aeroallergens are known to be airborne allergens that usually consist of airborne particles, the sources of which are mite feces and pollen grains [34], [35]; on the surface of SPM, well-known allergens like *Fel* d 1 (cat), *Can* f 1 (dog) and *Bet* v 1 (birch pollen) could attach, but *Der* p 1 could not [36], although these allergens were detected in an indoor environment and there is no evidence to show the contribution of these allergens in an outdoor environment consisting of PM_10_ or PM_2.5_. In SPM and PM_2.5_, diesel exhaust particles (DEP) are an important component as an adjuvant in OVA-induced airway allergic reactions [6], [11]. DEP may have been contained in the Pre in this study. Therefore, it is considered that DEP in Pre may act as an adjuvant for natural allergens or aeroallergens described above. However, intratracheal instillation of DEP in NC/Nga mice weekly for 6 weeks could elicit increases in neutrophils and cytokine levels such as IL-4 and IL-5 [37]. In this study, Sup plus Pre did not induce IL-4 and IL-5, but induced IL-13 and eotaxin 1. Moreover, DEP could not elicit the secretion of IL-1β by inflammasome complex [23]. From the changes of cytokines and chemokines, the contribution of DEP may not be large in this study. However, the insoluble fraction of Pre may function as an adjuvant for the allergic protein in soluble fraction (Sup). In this study, the most important evidence is that the reconstituted PM_2.5_ from the soluble and insoluble fractions was allergic. On the basis of the calculation of protein content in the soluble fraction, 50 μg of protein was attached in 200 μg of Pre. Therefore, we assume the reconstituted PM2.5 is natural and PM2.5 itself is allergenic.

The presence of eosinophils is a characteristic feature of allergic asthma and is associated with the severity and AHR. Many studies have shown the importance of eosinophils as major effector cells in allergic AHR [38]. The elevation of eosinophils in BAL was supported by the up-regulation of mRNA of eotaxin 1, responsible for the expression of eosinophils. Moreover, Th2 cytokines such as IL-4, Il-5 and IL-13 are crucial for the pathogenesis of allergic asthma [15]. Up-regulation of mRNA for IL-13 in this study showed the involvement of IL-13 in the elevation of AHR.

The nucleotide-binding domain and leucine-rich repeat protein 3 (NLRP3), adaptor protein apoptosis-associated speck-like protein (ASC) and inactive caspase-1 forming complex, the so-called inflammasome, respond to infection and intracellular danger signals such as reactive oxygen species (ROS), ATP and uric acid crystals. Autoactivation of caspase-1 leads to the cleavage of immature pro-IL-1β to mature active IL-1β. Secreted IL-1β is said to be associated with atherosclerosis, diabetes, obesity, gout and autoimmune disease [26]. Airway epithelial NLRP3 inflammasome-mediated immune responses were demonstrated to occur due to urban PM [28]. PM_10_ activated membrane TLRs and the inflammasome NLRP3 in human monocyte cell line (THP-1)-derived macrophages [27]. However, diesel exhaust particles (DEP) could not cause a response of generating IL-1β in THP-1 cells [22]. The involvement of IL-1β in an OVA-induced asthma model was demonstrated in attenuated airway hypersensitivity response (AHR) in IL-1α/β knockout mice [39] and reduced airway eosinophilic inflammation and goblet cell hyperplasia in mice lacking the IL-1β receptor [40]. Moreover, the involvement of inflammasome in an OVA-induced asthma model was shown by the increased generation of IL-1β in broncho-epithelial cells into the lumen of the bronchus [28]. These findings support our result of the increase in IL-1β in BALF and up-regulation of IL-1 mRNA in lung tissue. However, previously, there was no evidence of an *in vivo* study showing the involvement of NLRP-3-linked inflammasome in mice with PM-induced asthma. In this study, the involvement of inflammasome in PM_2.5_-induced asthma was demonstrated for the first time using NC/Nga mice.

Concerning the eliciting effect of PM_2.5_ for airway allergic reactions in an animal model, a few findings have been made. Sup and Pre in this study contained various metals. The metal and sulfate composition of residual oil fly ash contributed to airway hypersensitivity and lung injury in rats [12] and ambient urban Baltimore PM induced AHR and inflammation in mice [13]. The contribution of metals in PM-induced pulmonary injury was associated with an increase in eosinophils [8]. However, the mechanism of urban Baltimore PM-induced AHR in mice has yet to be resolved. Although the distribution of metals between water-soluble Sup and insoluble Pre was not equal, metals contained in both Sup and Pre were not ruled out as a major factor.

In an in vivo experiment involving PM exposure, contaminating bacterial lipopolysaccharide (LPS) should be considered as a factor affecting lung toxicity and asthma experiments. LPS reduced asthma by transmembrane Toll-like receptor 4 (TLR4)-mediated mechanisms [41]. LPS stimulates inflammasome in the lung as well as the heart [42]. LPS is known as a potent inducer of IFN-γ generated by the Th1-type immune system [43]. Since IFN-γ and IL-1β were not increased in the mouse group of Sup, there was considered to be little effect of LPS contained in PM_2.5_ in this study.

As a histopathological finding, intracellular localization of PM particles in macrophage-like cells was observed near the inflammatory granulation. However, it is not known what mechanisms are involved in the entry of PM_2.5_ particles into macrophages, or what interactions are involved between allergic reaction and the synergic action of PM_2.5_.

In this study, we demonstrated for the first time that ambient PM_2.5_ could elicit airway allergic reaction involving inflammasome mechanisms by the synergistic action of soluble factor and insoluble particles in NC/Nga mice. Our results suggest that some natural allergens in the soluble fraction of PM_2.5_ may contribute to allergic airway reaction. Therefore, in future, investigations should be undertaken to determine which soluble factors contribute and how inflammasomes are involved in the synergistic action of Sup and Pre in allergic airway reaction.
