# Correction: Allergic Airway Inflammation by Nasal Inoculation of Particulate Matter (PM_2.5_) in NC/Nga Mice

**DOI:** 10.1371/journal.pone.0103465

**Published:** 2014-07-17

**Authors:** The PLOS ONE Staff

There are errors in the Materials and Methods section of this article.

Under the subheading “Chemicals”, the first sentence is incorrect. The correct sentence is: Oligonucleotide primers for real-time PCR were synthesized by Invitrogen (Waltham, MA, USA).

Under the subheading “Separation of Particulate Matter Fractions”, the second sentence is incorrect. The correct sentence is: Contaminated quartz fibers were filtrated with an 11 µm filter (nylon net filter NY11, Merck Millipore, Billerica, MA, USA) and the filtrate was concentrated by vacuum freeze-drying.
